# Practical one-dimensional measurements of age-related brain atrophy are validated by 3-dimensional values and clinical outcomes: a retrospective study

**DOI:** 10.1186/s12880-016-0136-x

**Published:** 2016-04-26

**Authors:** C. Michael Dunham, Albert J. Cook, Alaina M. Paparodis, Gregory S. Huang

**Affiliations:** Trauma/Critical Care Services, St. Elizabeth Youngstown Hospital, 1044 Belmont Avenue, Youngstown, OH 44501 USA; Division of Radiology, St. Elizabeth Youngstown Hospital, 1044 Belmont Avenue, Youngstown, OH 44501 USA

**Keywords:** Brain atrophy, Traumatic intracranial hemorrhage, CT imaging

## Abstract

**Background:**

Age-related brain atrophy has been represented by simple 1-dimensional (1-D) measurements on computed tomography (CT) for several decades and, more recently, with 3-dimensional (3-D) analysis, using brain volume (BV) and cerebrospinal fluid volume (CSFV). We aimed to show that simple 1-D measurements would be associated with 3-D values of age-related atrophy and that they would be related to post-traumatic intracranial hemorrhage (ICH).

**Methods:**

Patients ≥60 years with head trauma were classified with central atrophy (lateral ventricular body width >30 mm) and/or cortical atrophy (sulcus width ≥2.5 mm). Composite atrophy was the presence of central or cortical atrophy. BV and CSFV were computed using a Siemens Syngo workstation (VE60A).

**Results:**

Of 177 patients, traits were age 78.3 ± 10, ICH 32.2 %, central atrophy 39.5 %, cortical atrophy 31.1 %, composite atrophy 49.2 %, BV 1,156 ± 198 mL, and CSFV 102.5 ± 63 mL. CSFV was greater with central atrophy (134.4 mL), than without (81.7 mL, *p* < 0.001). BV was lower with cortical atrophy (1,034 mL), than without (1,211 mL; *p* < 0.001). BV was lower with composite atrophy (1,103 mL), than without (1,208 mL; *p* < 0.001). CSFV was greater with composite atrophy (129.1 mL), than without (76.8 mL, *p* < 0.001). CSFV÷BV was greater with composite atrophy (12.3 %), than without (6.7 %, *p* < 0.001). Age was greater with composite atrophy (80.4 years), than without (76.3, *p* = 0.006). Age had an inverse correlation with BV (*p* < 0.001) and a direct correlation with CSFV (*p* = 0.0002) and CSFV÷BV (*p* < 0.001). ICH was greater with composite atrophy (49.4 %), than without (15.6 %; *p* < 0.001; odds ratio = 5.3). BV was lower with ICH (1,089 mL), than without (1,188 mL; *p* = 0.002). CSFV÷BV was greater with ICH (11.1 %), than without (8.7 %, *p* = 0.02). ICH was independently associated with central atrophy (*p* = 0.001) and cortical atrophy (*p* = 0.003).

**Conclusions:**

Simple 1-D measurements of age-related brain atrophy are associated with 3-D values. Clinical validity of these methods is also supported by their association with post-injury ICH. Intracranial 3-D software is not available on many CT scanners and can be cumbersome, when available. Simple 1-D measurements, using the study methodology, are a practical method to objectify the presence of age-related brain atrophy.

## Background

Although brain atrophy has been touted for several decades as a risk for post-traumatic intracranial hemorrhage (ICH), our group recently provided the only published evidence validating this notion [1]. Specifically, we showed that 1-dimensional (1-D) estimates of atrophy on brain computed tomography (CT) have an association with the rate of ICH. For several decades, investigators have used 1-D measurements to indicate the presence of age-associated brain atrophy. The primary representations of brain atrophy have been lateral ventricular enlargement [2–5] and cortical sulcal widening [2, 3, 6–12]. At least six investigations of non-demented patients have indicated that with advancing age, the ventricular or intracranial cerebrospinal fluid (CSF) volume increases [13–17] and the brain volume decreases [13–15, 17, 18]. We performed brain and CSF 3-dimensional (3-D) measurements on patients described in our previous report. We hypothesized that 1-D and 3-D brain CT measurements of age-related brain atrophy would have an association. We also hypothesized that 1-D and 3-D measurements of brain atrophy would be related to post-traumatic ICH.

## Methods

### Patient inclusion

Inclusion criteria were age ≥ 60 years, fall from standing height or motor vehicle crash, physical evidence of head trauma (i.e., facial fracture, skull fracture, scalp soft tissue injury, facial soft tissue injury, or cervical spine injury), and trauma center admission. Brain CT images were obtained when the patient arrived at the trauma center and stored in the regional picture archiving and communication system (PACS) as digital imaging and communications in medicine (DICOM) files. ICH was determined as absent or present, according to the radiology report, which was confirmed by the first author. For patients with ICH producing major brain compression (midline shift, lateral ventricular compression, or mesencephalic cistern compression), patients were included, only if they had a CT without brain compression within 6 months prior to their injury.

### 1-D measurements

**Fig. 1:**
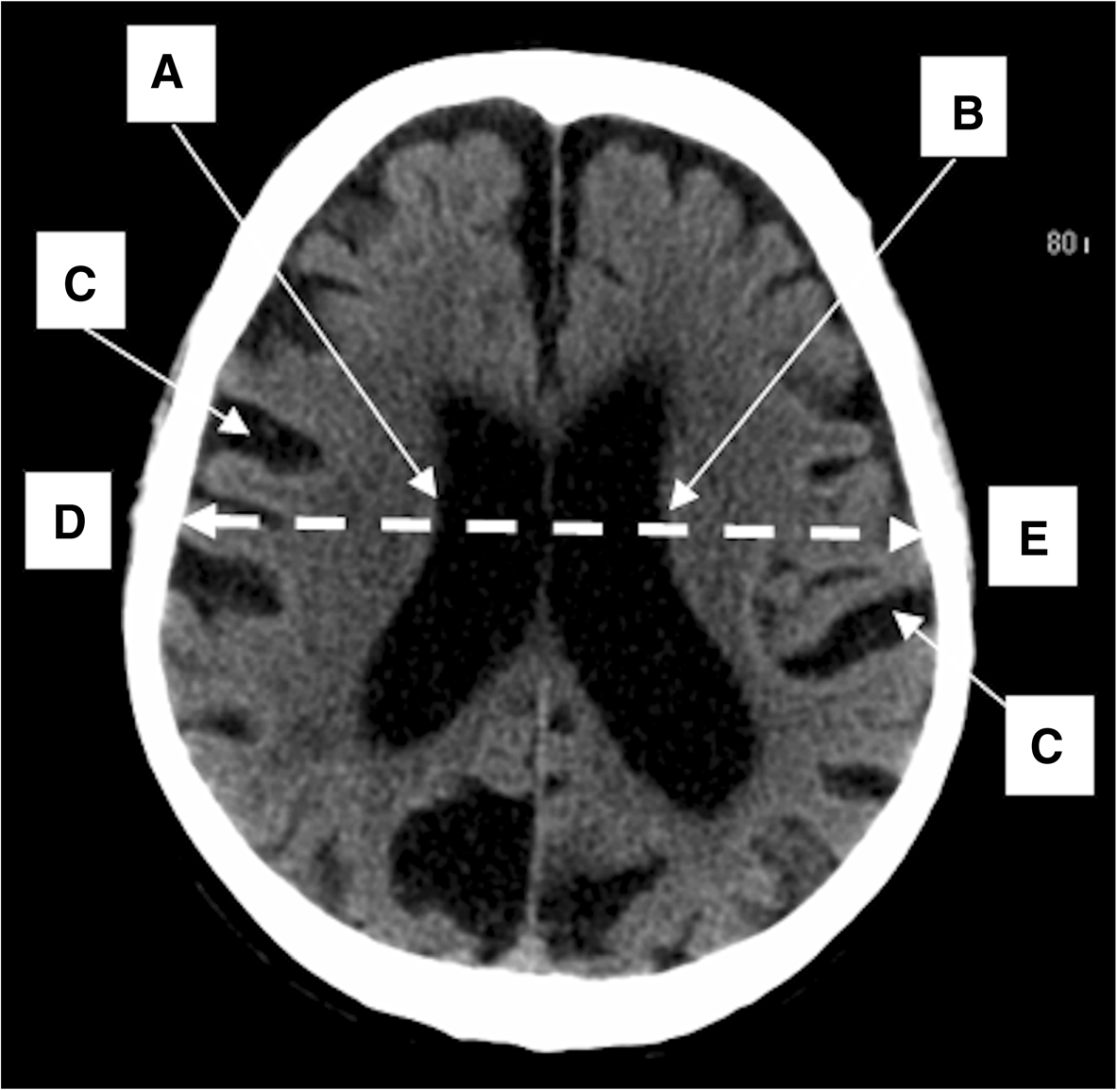
1-Dimensional Measurements. Transverse distance *“A-to-B”* is the maximum lateral ventricle body width; *“C”* is ≥ 2 cortical sulci, measuring ≥2.5 mm each; and transverse distance *“D-to-E”* is the intracranial width (at the level of *“A-to-B”*)

The DICOM files were retrieved from PACS, and the images were opened in a viewer for evaluating brain CT images. Axial views of the brain CT were reviewed to locate and measure the maximal transverse width of the left and right lateral ventricular bodies (i.e., the ventricular width). The brain width (i.e., the right and left transverse distances from the cortical surface to the ipsilateral ventricular margin) was measured at the axial level of the maximal lateral ventricular body width. The intracranial width was computed as the sum of the brain width and the ventricular width. The cortical sulcus width was assessed at the level of the maximal lateral ventricular body width. Cortical atrophy was considered present whenever two or more sulci each had a width ≥2.5 mm with a decreased adjacent gyral width. Central atrophy was considered to be present whenever the lateral ventricular body width was >30 mm. Composite atrophy was the presence of cortical atrophy, central atrophy, or both. The principal 1-D measurements are depicted in Fig. 1.

### 3-D measurements

**Fig. 2:**
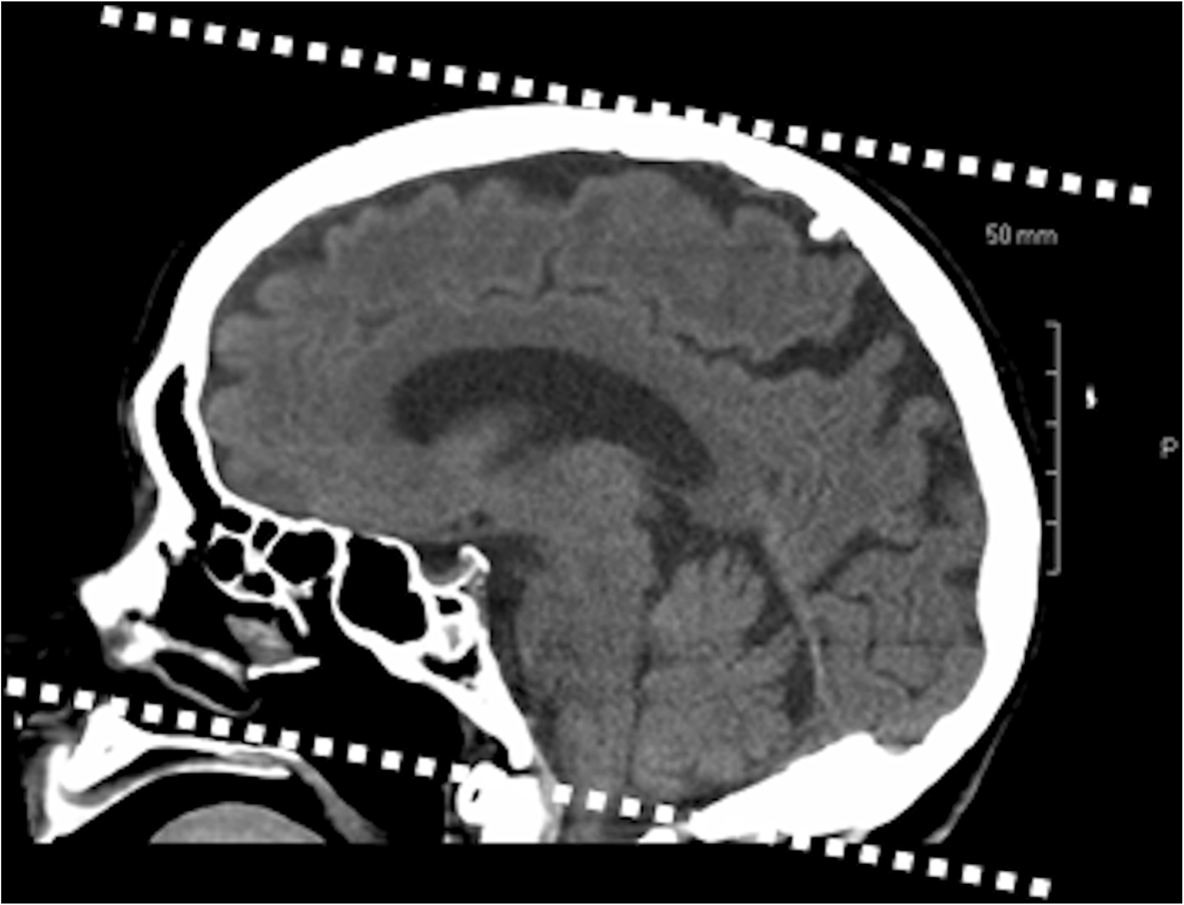
3-Dimensional Measurements. The 3-dimensional measurements were obtained from the level of the skull vertex and caudal to the level of the foramen magnum

**Fig. 3:**
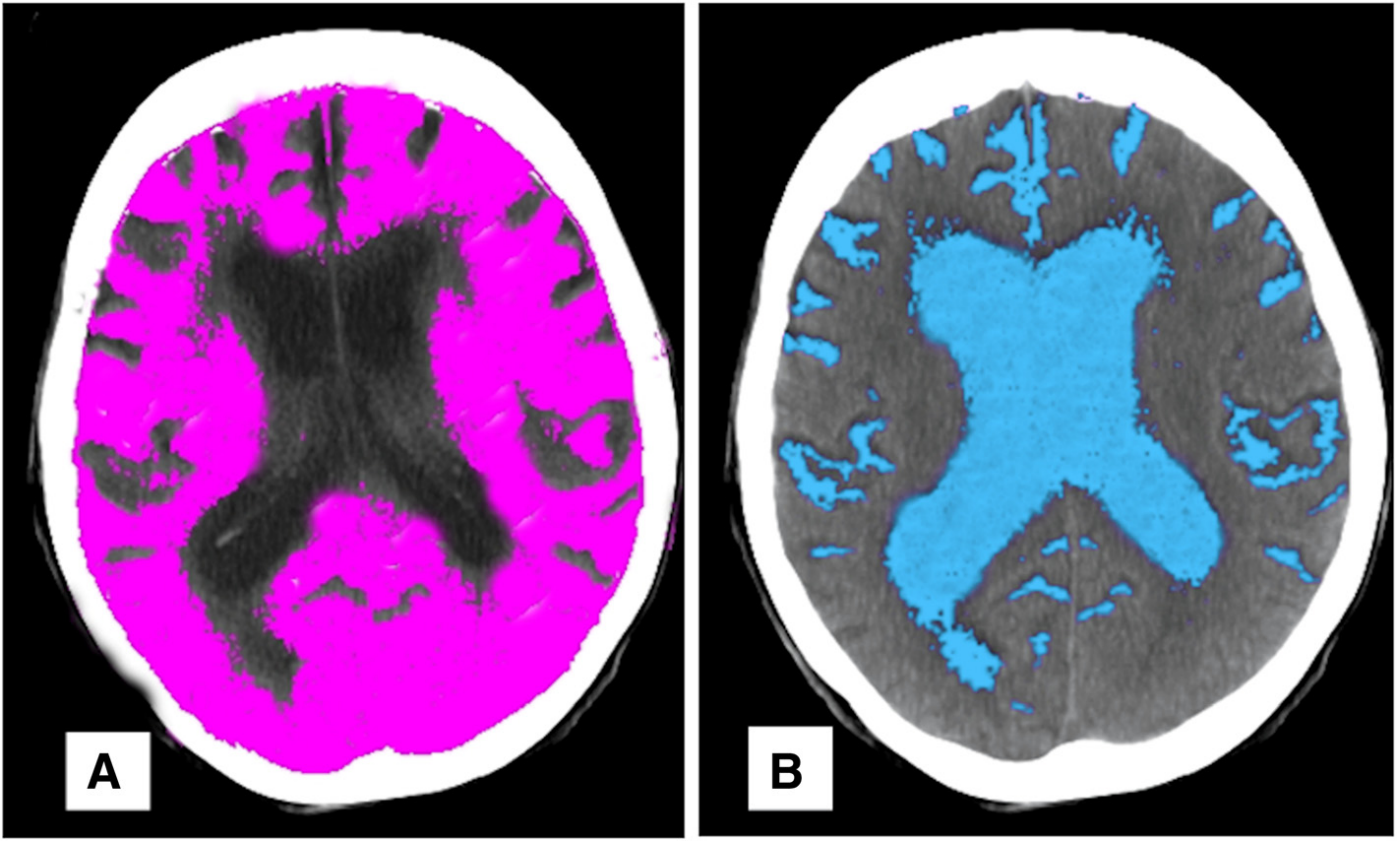
3-D Threshold Fields. **a** highlighting of brain parenchyma (Hounsfield range 22-55); **b** highlighting of CSF (Hounsfield range 0-15)

**Fig. 4:**
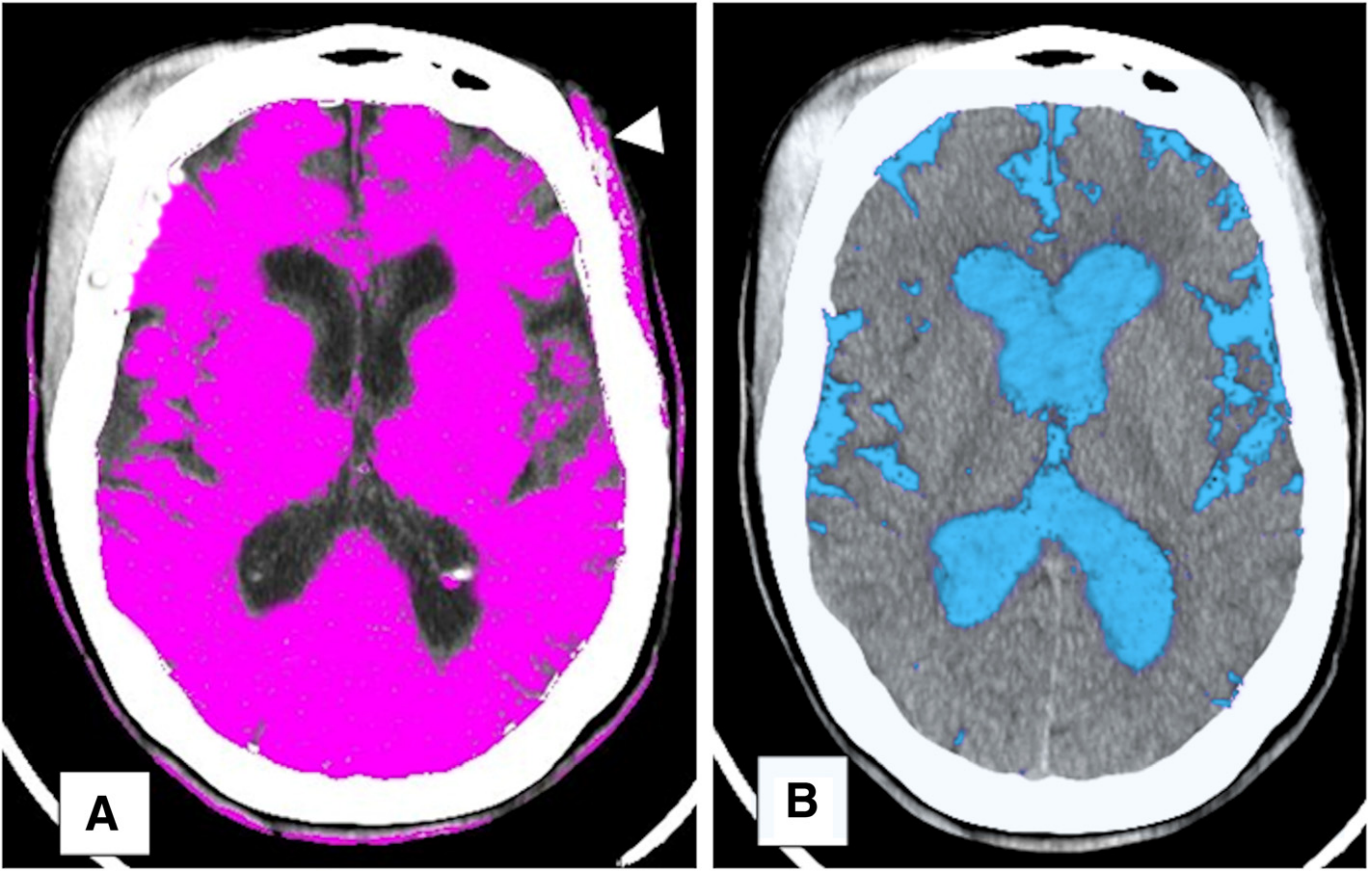
3-D Threshold Fields. **a** highlighting of brain parenchyma (Hounsfield range 22-55); **b** highlighting of CSF (Hounsfield range 0–15). Brain volume contamination in patient with extra-cranial hematoma (**a**: *white arrow*)

The DICOM files, produced by a 64 slice GE LightSpeed VCT CT system (Milwaukee, WI), were retrieved from PACS and migrated into the Siemens syngo.via platform (Siemens AG Healthcare Sector, Erlangen, Germany). The DICOM files were then imported into a Siemens Syngo multimodality workplace workstation (VE60A) and converted to a readable format. The CSF volume was computed by including pixels with a Hounsfield range of 0–15 and the brain volume was calculated from pixels with a Hounsfield range of 22–55. The Hounsfield ranges were selected by the second author, a neuroradiologist, by observing the colored areas of tissues that would be included on the CT images, when various Hounsfield values were used. Intracranial slices included in the 3-D computation were those from the cranial vertex to the foramen magnum (Fig. 2). An example of typical colored areas of included tissue is in Fig. 3 and an infrequent instance with contamination is in Fig. 4. The second author, a neuroradiologist, and the third author, a certified CT technician, supervised the 3-D computation process.

### Patient exclusion

Because patients with ICH producing major brain compression would not permit accurate 1-D and 3-D measurements, they were excluded, unless a CT scan without brain compression was archived in the PACS within the 6 months prior their injury. Patients were excluded if they met the age and injury criteria, yet the DICOM files would not migrate into the Siemens syngo.via platform.

### Ancillary data

Hospital admission neurologic function was a dichotomization of the Glasgow Coma Scale score. A score of 13-15 represented normal function or minor dysfunction. A score of 3-12 indicated major admission neurologic dysfunction. Hospital death status, age, gender status, and 3-month outcomes were also available. A 3-month outcome was considered to be good, if there was normal or minimally abnormal neurologic function, and bad, if death had occurred or there was severe neurologic dysfunction (vegetative state or coma). Also available in our reliable database was a pre-existing medical condition status for each patient. Conditions included presence or absence of a pre-hospital admission history for dementia, anemia, hypertension, cardiac disease, anti-thrombotic agent use, diabetes mellitus, pulmonary disease, and cerebrovascular accident.

### Statistical analysis

Data were entered into a Microsoft Excel® 2010 spreadsheet (Microsoft Corp., Redmond, WA) and imported into SAS System for Windows, release 9.2 (SAS Institute Inc., Cary, NC), for statistical analyses. For the continuous variable cohort data, standard deviation was used to complement the mean. Pearson correlation analysis was performed for assessing association between two continuous variables. Periodically and for simplification of presentation, cortical sulcus width ≥2.5 mm was expressed as a dichotomous (dummy) variable for correlation analysis. In this circumstance, a one represented its presence and a zero its absence. Wilcoxon Rank Sum test was used to compare continuous data between two groups. Fischer’s exact test was used to assess the relationship of two dichotomous variables. Multivariate logistic regression analysis was performed to assess the relationship between a dependent variable that was dichotomous and potential independent variables. The level of significance was considered *p* < 0.05.

## Results

The original cohort included 198 patients with physical evidence of head trauma, age ≥60 years, and an ICH rate of 36.4 % (*n* = 72). Because ICH created extensive compression and a pre-injury CT within 6 months was unavailable, 1-D estimates of atrophy were not measured in 6 patients. The ICH rate for the 192 patients was 34.9 % (*n* = 67). Of the 192 patients, we did not perform intracranial 3-D estimates of atrophy in 15 (7.8 %), because the DICOM file could not be retrieved from PACS or they failed to migrate into the Siemens workspace. The ICH rate for these 177 patients was 32.2 % (*n* = 57) and the ICH rates for these three groups (36.4 %, 34.9 %, and 32.2 %) were similar (*p* = 0.7021). One hundred seventy-seven patients comprise the subset that underwent subsequent analysis. The patients’ age was 78.3 ± 10.0 years (range, 60–99 years) and the Glasgow Coma Scale score was 14.3 ± 2.0 (range, 3–15). Of the 57 patients with ICH included in the analyses, 7 (12.3 %) had major brain compression.

### 1-D and 3-D associations

The intracranial 1-D and 3-D outcomes are shown in Table 1.

**Table 1:** Intracranial 1-dimensional and 3-dimensional outcome

	Value
1-Dimensional Measurements:	
Ventricular Width (mm)	31.2 **±** 5.6
Cortical Sulcus Width ≥2.5 mm	31.1 %
Brain Width (mm)	90.6 **±** 8.5
Ventricular Width ÷ Brain Width (%)	34.8 **±** 7.4
Ventricular Width ÷ Intracranial Width (%)	25.6 **±** 4.0
3-Dimensional Measurements:	
CSF Volume (mL)	102.5 **±** 63
Brain Volume (mL)	1156.4 **±** 198
Intracranial Volume (mL)	1258.9 **±** 185
CSF Volume ÷ Brain Volume (%)	9.5 **±** 6.6
CSF Volume ÷ Intracranial Volume (%)	8.3 **±** 5.2

Age had a negative association with the brain width and brain volume and a positive correlation with a cortical sulcus width ≥2.5 mm, the ventricular width ÷ brain width, ventricular width ÷ intracranial width, CSF volume, CSF volume ÷ brain volume, and CSF volume ÷ intracranial volume; and a trend for a positive association with the ventricular width (Table 2). Age was ≥75 years in 65.0 % (115/177) of the patients and 60-74 years in 35.0 % (62/177). The ventricular width was greater for age ≥75 years (31.9 ± 5.4 mm), when compared to younger patients (29.9 ± 5.8 mm; Wilcoxon Rank Sum test: *p* = 0.0217). The cortical atrophy rate was increased for age ≥75 years (42.6 % [49/115]), when compared to younger patients (9.7 % [6/62]; Fischer’s exact test: *p* < 0.0001). CSF volume was greater for age ≥75 years (116 ± 60 mL), when compared to younger patients (78 ± 59 mL; Wilcoxon Rank Sum test: *p* < 0.0001). Brain volume was lower for age ≥75 years (1086 ± 164 mL), when compared to younger patients (1288 ± 187 mL; Wilcoxon Rank Sum test: *p* < 0.0001).

**Table 2:** Age Correlations

	r-Value	P-Value
1-Dimensional Measurements:		
Ventricular Width (mm)	+0.13	0.0937
Cortical Sulcus Width ≥2.5 mm	+0.35	<0.0001
Brain Width (mm)	−0.28	0.0001
Ventricular Width ÷ Brain Width (%)	+0.22	0.0041
Ventricular Width ÷ Intracranial Width (%)	+0.23	0.0017
3-Dimensional Measurements:		
Brain Volume (mL)	−0.55	< 0.0001
CSF Volume (mL)	+0.28	0.0002
CSF Volume ÷ Brain Volume (%)	+0.36	< 0.0001
CSF Volume ÷ Intracranial Volume (%)	+0.38	< 0.0001

Pearson correlation analysis was used for statistical assessment; Cortical Sulcus Width ≥2.5 mm was represented as a dichotomous (dummy) variable

The ventricular width correlations were negative with the brain volume and positive with the CSF volume, CSF volume ÷ brain volume, and CSF volume ÷ intracranial volume (Table 3). The cortical sulcus width ≥2.5 mm correlations were negative with the brain volume and positive with the CSF volume, CSF volume ÷ brain volume, and CSF volume ÷ intracranial volume (Table 3). The brain width correlations were negative with the CSF volume, CSF volume ÷ brain volume, and CSF volume ÷ intracranial volume and positive with the brain volume (Table 3). The ventricular width ÷ brain width correlations were negative with the brain volume and positive with the CSF volume, CSF volume ÷ brain volume, and CSF volume ÷ intracranial volume (Table 3). The ventricular width ÷ intracranial width correlations were negative with the brain volume and positive with the CSF volume, CSF volume ÷ brain volume, and CSF volume ÷ intracranial volume (Table 3).

**Table 3:** 1-Dimensional and 3-Dimensional Correlations

	CSF volume	Brain volume	CSF volume ÷ Brain volume	CSF volume ÷ Intracranial volume
Ventricular Width (mm)	*r* = 0.46	*r* = -0.13	*r* = 0.42	*r* = 0.43
	*p* < 0.0001	*p* = 0.0803	*p* < 0.0001	*p* < 0.0001
Cortical Sulcus Width ≥2.5 mm	*r* = 0.45	*r* = -042	*r* = 0.50	*r* = 0.50
	*p* < 0.0001	*p* < 0.0001	*p* < 0.0001	*p* < 0.0001
Brain Width (mm)	*r* = -0.16	*r* = 0.32	*r* = -0.20	*r* = -0.21
	*p* = 0.0392	*p* < 0.0001	*p* = 0.0091	*p* = 0.0057
Ventricular ÷ Brain Width	*r* = 0.45	*r* = -0.24	*r* = 0.44	*r* = 0.45
	*p* < 0.0001	*p* = 0.0016	*p* < 0.0001	*p* < 0.0001
Ventricular ÷ Intracranial Width	*r* = 0.46	*r* = -0.25	*r* = 0.44	*r* = 0.46
	*p* < 0.0001	*p* = 0.0008	*p* < 0.0001	*p* < 0.0001

Pearson correlation analysis was used for statistical assessment; Cortical Sulcus Width ≥2.5 mm was represented as a dichotomous (dummy) variable

Central atrophy (lateral ventricular body width >30 mm) occurred in 39.5 % and was associated with a reduction in brain volume and an increase in the CSF volume, CSF volume ÷ brain volume, and CSF volume ÷ intracranial volume (Table 4). Central atrophy was more frequent with a CSF volume ≥100 mL (57.1 % [44/77]), than with a CSF volume <100 mL (26.0 % [26/100]; Fisher’s exact test: *p* < 0.0001; odds ratio [OR= = 3.8). Cortical atrophy (cortical sulcus width ≥2.5 mm) occurred in 31.1 % and was associated with a reduction in the brain volume and an increase in the CSF volume, CSF volume ÷ brain volume, and CSF volume ÷ intracranial volume (Table 5). Cortical atrophy was more frequent with a brain volume <1,100 mL (51.3 % [39/76]), than with a brain volume ≥1,100 mL (15.8 % [16/101]; Fisher’s exact test: *p* < 0.0001; OR = 5.6). Composite atrophy (ventricular or cortical atrophy) occurred in 49.2 % and was associated with a reduction in the brain volume and an increase in the CSF volume, CSF volume ÷ brain volume, and CSF volume ÷ intracranial volume (Table 6). Composite atrophy was more frequent with a CSF volume ÷ brain volume ≥9.0 % (68.8 % [53/77]), than with a CSF volume ÷ brain volume <9.0 % (34.0 % [34/100]; Fisher’s exact test: *p* < 0.0001; OR = 4.3). Composite atrophy was more frequent with a CSF volume ÷ intracranial volume ≥10.0 % (73.7 % [42/57]), than with a CSF volume ÷ intracranial volume <10.0 % (37.5 % [45/120]; Fisher’s exact test: *p* < 0.0001; OR = 4.7).

**Table 4:** Central Atrophy Correlations

	No central atrophy	Central atrophy	*P*-Value
Number	107 (60.5 %)	70 (39.5 %)	
Brain Volume (mL)	1184 ± 201	1114 ± 186	0.0196
CSF Volume (mL)	81.7 ± 46.2	134.4 ± 70.6	<0.0001
CSF Volume ÷ Brain Volume (%)	7.3 ± 5.0	12.7 ± 7.4	<0.0001
CSF Volume ÷ Intracranial Volume (%)	6.7 ± 4.0	10.9 ± 5.7	<0.0001

Wilcoxon Rank Sum test was used for statistical assessment

**Table 5:** Cortical Atrophy Correlations

	No cortical atrophy	Cortical atrophy	*P*-Value
Number	122 (68.9 %)	55 (31.1 %)	
Brain Volume (mL)	1211 ± 188	1034 ± 162	<0.0001
CSF Volume (mL)	83.7 ± 49.2	144.3 ± 86.8	<0.0001
CSF Volume ÷ Brain Volume (%)	7.3 ± 4.8	14.4 ± 7.4	<0.0001
CSF Volume ÷ Intracranial Volume (%)	6.6 ± 3.9	12.2 ± 5.5	<0.0001

Wilcoxon Rank Sum test was used for statistical assessment

**Table 6:** Composite Atrophy Correlations

	No composite atrophy	Composite atrophy	*P*-Value
Number	90 (50.8 %)	87 (49.2 %)	
Brain Volume (mL)	1208 ± 194	1103 ± 188	<0.0001
CSF Volume (mL)	76.8 ± 42.7	129.1 ± 68.5	<0.0001
CSF Volume ÷ Brain Volume (%)	6.7 ± 4.2	12.3 ± 7.4	<0.0001
CSF Volume ÷ Intracranial Volume (%)	6.2 ± 3.6	10.6 ± 5.6	<0.0001

Wilcoxon Rank Sum test was used for statistical assessment

### ICH associations

ICH was associated with the ventricular width, ventricular width ÷ brain width, ventricular width ÷ intracranial width, central atrophy, cortical atrophy, and composite atrophy (Table 7). ICH was also associated with a decreased brain volume and an increased CSF volume, CSF volume ÷ brain volume, and CSF volume ÷ intracranial volume; however, age and gender had no correlation (Table 7). The rate of ICH progressively increased with central atrophy or cortical atrophy or both (Table 8). Multivariate logistic regression analysis demonstrated that ICH had an independent association with central atrophy (*p* = 0.0011) and cortical atrophy (*p* = 0.0029).

**Table 7:** Intracranial Hemorrhage Correlations

	No ICH	ICH	*P*-Value
Number	120 (67.8 %)	57 (32.2 %)	
1-Dimensional Measurements:			
Ventricular Width (mm)	30.1 ± 5.3	33.5 ± 5.4	0.0001
Brain Width (mm)	90.6 ± 8.3	90.5 ± 9.1	0.9921
Ventricular Width ÷ Brain Width (%)	33.6 ± 6.9	37.5 ± 7.6	0.0009
Ventricular Width ÷ Intracranial Width (%)	24.9 ± 3.8	27.0 ± 4.0	0.0008
Central Atrophy	33 (27.5 %)	37 (64.9 %)	<0.0001
Cortical Atrophy	24 (20.0 %)	31 (54.4 %)	<0.0001
Composite Atrophy	44 (36.7 %)	43 (75.4 %)	<0.0001
3-Dimensional Measurements:			
CSF Volume (mL)	97.0 ± 55	114.2 ± 75	0.0875
Brain Volume (mL)	1188 ± 201	1089 ± 172	0.0017
CSF Volume ÷ Brain Volume (%)	8.7 ± 5.6	11.1 ± 8.0	0.0186
CSF Volume ÷ Intracranial Volume (%)	7.7 ± 4.5	9.6 ± 6.2	0.0254
Age (years)	77.7 ± 9.9	79.8 ± 10.0	0.1861
Female	61 (50.8 %)	26 (45.6 %)	0.5163

Wilcoxon Rank Sum test was used for statistical analysis of continuous data and Fischer’s exact test was used for dichotomous data

**Table 8:** Intracranial hemorrhage rates according to the patients’ cortical and central atrophy status

Cortical atrophy	Central atrophy	Patient no.	ICH rate
No	No	90	15.6 %
Yes	No	17	35.3 %
No	Yes	32	37.5 %
Yes	Yes	38	65.8 %

Of the 177 patients, 11 (6.2 %) had major admission neurologic dysfunction and 165 (93.8 %) did not. The ICH rate was greater in patients with major neurologic dysfunction (63.6 % [7/11]), when compared to those without (30.1 % [50/165]; Fischer’s exact test: *p* = 0.0212; OR = 4.1). The ICH with compression was also higher for patients with major dysfunction (27.3 % [3/11]), when compared to those without (2.4 % [4/165]; Fischer’s exact test: *p* < 0.0001; OR = 15.2). ICH was more frequent in dying patients (75.0 % [6/8]), when compared those surviving hospitalization (30.2 % [51/160]; Fischer’s exact test: *p* = 0.0078). ICH with compression was also more frequent in dying patients (37.5 % [3/8]), when compared those surviving hospitalization (2.4 % [4/160]; Fischer’s exact test: *p* < 0.0001; RR = 1.7).

### Ancillary associations with 1-D and 3-D measurements

Relative to females, males were found to have increased brain width, increased intracranial width, increased brain volume, increased intracranial volume, and decreased CSF volume ÷ intracranial volume (Table 9).

**Table 9:** Cranial measurements by gender status

	Male	Female	*P*-value
Number	90 (50.8 %)	87 (49.2 %)	
1-Dimensional Measurements:			
Ventricular Width (mm)	31.9 ± 6.2	30.5 ± 4.8	0.0923
Coritcal Sulcus Width ≥2.5 mm	29.9 %	33.3 %	0.5230
Brain Width (mm)	93.1 ± 8.5	87.9 ± 7.8	<0.0001
Intracranial Width (mm)	125 ± 10	118 ± 9	<0.0001
Ventricular Width ÷ Brain Width (%)	34.6 ± 8.0	35.0 ± 6.8	0.7551
Ventricular Width ÷ Intracranial Width (%)	25.5 ± 4.3	25.7 ± 3.6	0.6562
3-Dimensional Measurements:			
CSF volume (mL)	102 ± 69	104 ± 55	0.8400
Brain volume (mL)	1277 ± 179	1032 ± 125	<0.0001
Intracranial volume (mL)	1379 ± 159	1135 ± 116	<0.0001
CSF Volume ÷ Brain Volume (%)	8.5 ± 6.8	10.4 ± 6.2	0.0532
CSF Volume ÷ Intracranial Volume (%)	7.5 ± 5.3	9.2 ± 4.9	0.0331

Wilcoxon Rank Sum test was used for statistical analysis of continuous data and Fischer’s exact test was used for dichotomous data

Patients with dementia had more cortical atrophy, reduced brain width, increased ventricular ÷ brain width, increased ventricular ÷ intracranial width, increased CSF volume, reduced brain volume, increased CSF volume ÷ brain volume, and increased CSF volume ÷ intracranial volume (Table 10). Dementia rates were similar for patients with major admission neurologic dysfunction (9.1 % [1/11]) and those without (20.0 % [33/166]; Fischer’s exact test: *p* = 0.0886). Females had a higher rate of dementia (27.6 % [24/87]), when compared to males (11.1 % [10/90]; Fischer’s exact test: *p* = 0.0054).

**Table 10:** Cranial measurements by dementia status

	No dementia	Dementia	*P*-value
	143 (80.8 %)	34 (19.2 %)	
1-Dimensional Measurements:			
Coritcal Sulcus Width ≥2.5 mm	27.3 %	47.1 %	0.0250
Brain Width (mm)	91.5 ± 8.2	86.6 ± 8.7	0.0022
Ventricular ÷ Brain Width (%)	34.2 ± 7.0	37.3 ± 8.3	0.0262
Ventricular ÷ Intracranial Width (%)	25.3 ± 3.9	27.0 ± 4.2	0.0288
3-Dimensional Measurements:			
CSF Volume (mL)	96.4 ± 62.7	128.2 ± 55.2	0.0073
Brain Volume (mL)	1185.3 ± 190	1034 ± 186	<0.0001
Intracranial Volume (mL)	1282 ± 182	1163 ± 168	0.0007
CSF Volume ÷ Brain Volume (%)	8.6 ± 6.1	13.2 ± 7.3	0.0002
CSF Volume ÷ Intracranial Volume (%)	7.6 ± 4.9	11.3 ± 5.4	0.0001

Wilcoxon Rank Sum test was used for statistical analysis of continuous data and Fischer’s exact test was used for dichotomous data

Patients with pre-admission anemia had a higher CSF volume ÷ brain volume (*n* = 17; 14.4 %), when compared to those without anemia (*n* = 160; 8.9 %; Wilcoxon Rank Sum test: *p* = 0.0010). Patients with anemia also had higher ventricular width ÷ intracranial width (27.8 %), when compared to no anemia (25.4 %; Wilcoxon Rank Sum test: *p* = 0.0140). Patients with a cardiac history had a higher rate of cortical atrophy (39.7 % [29/73), when compared to no cardiac history (25.0 % [26/104]; Fisher’s exact test: *p* = 0.0373). As well, those with a cardiac history had a greater rate of composite atrophy (60.3 % [44/73), when compared to patients with no cardiac history (41.4 % [43/104]; Fisher’s exact test: *p* = 0.0130). Patients receiving anti-thrombotic agents also had a higher rate of composite atrophy (54.0 % [61/113]), when compared to those with no use (40.6 % [26/64]; Fisher’s exact test: *p* = 0.0886). A pre-admission history of diabetes mellitus, pulmonary disease, or cerebrovascular accident did not show any association with the 1-D or 3-D measurements.

Of the 168 patients discharged from the hospital, 137 (81.5 %) had a good outcome at 3-months, and 31 (18.5 %) had a bad outcome. Those with a bad outcome had a higher CSF volume ÷ brain volume (11.9 %), when compared to patients with a good outcome (9.0 %; Wilcoxon Rank Sum Test: *p* = 0.0258). The major admission neurologic dysfunction rate was greater in patients with a bad outcome (12.9 % [4/31]), when compared to those with a good outcome (1.5 % [2/137]; Fischer’s exact test: *p* = 0.0018). The rate of pre-hospital dementia was higher in those with a bad outcome (35.5 % [11/31]), when compared to those with a good outcome (16.1 % [22/137]; Fischer’s exact test: *p* = 0.0140). Multivariate logistic regression analysis showed that bad outcome was independently associated with major admission neurologic dysfunction (*p* = 0.0042) and higher CSF volume ÷ brain volume (*p* = 0.0125). Analysis also demonstrated that bad outcome was independently associated with major admission neurologic dysfunction (*p* = 0.0072) and dementia (*p* = 0.0123).

## Discussion

Relative to our mean population values, other investigators have found comparable results for the ventricular width [19], ventricular width ÷ intracranial width [3–5, 7], CSF volume [13, 18, 20], brain volume [13, 18, 20], intracranial volume [16, 18, 20, 21], CSF volume ÷ brain volume [13, 18], and CSF volume ÷ intracranial volume [18, 21]. Threshold imperfections were minor and infrequent. The similarity of our values, to those published by other researchers, suggests that our methodological techniques were valid and appropriate.

### Age associations

We demonstrated multiple correlations of age with the 1-D and 3-D intracranial measurements. We showed that the lateral ventricular body width increased with age, a finding also noted by Meese [19]. Of relevance, age has also been shown to be associated with an increased lateral ventricular volume [17, 22], total ventricular volume [14–16], and lateral ventricular volume ÷ intracranial volume [22]. We found that the ventricular width ÷ brain width increased with progressive age, a finding also noted by Earnest [3]. Our association of ventricular width ÷ intracranial width with age has been corroborated by others [5]. Other investigators have also substantiated our finding that the brain volume decreases with advancing age [13, 14, 17, 18]. Similarly, Matsumae [18] and Gur [13] have also noted an increase in the CSF volume with progressive aging. Finally, others have validated our finding that increasing age is associated with an increase in the CSF volume ÷ intracranial volume [18, 21]. Our multiple correlations with age, with corroboration by other investigators, imply that our measurements are reliable estimates.

### 1-D and 3-D measurement associations

Multiple associations were noted between the relevant 1-D and 3-D measurements. In particular, logical correlations existed between the brain volume and cortical sulcus width and the brain width. Further, the CSF volume had a statistical and rational correlation with the ventricular width. Finally, the derivative 1-D variables correlated with their logically paired counterpart, e.g., the ventricular width ÷ intracranial width and CSF volume ÷ intracranial volume. These logical correlations support the validity of the 1-D and 3-D methods utilized to compute the dimensions. Although we found no publication in the literature that has attempted to correlate 1-D and 3-D measurements, in terms of age-related intracranial structures, the associations were reasonably expected.

We found that central atrophy (i.e., lateral ventricular body width >30 mm) was associated with a reduction in the brain volume and an increase in the CSF volume, CSF volume ÷ brain volume, and CSF volume ÷ intracranial volume. Previous investigators have concluded that age-associated brain atrophy is manifested by an increased ventricular size [5, 13–20, 22], CSF volume [13, 14, 18, 20], and CSF volume ÷ intracranial volume [18, 21] and decreased brain volume [13, 15, 17, 18, 20]. The present study findings and existing literature indicate that central atrophy is a valid indication of age-associated brain atrophy.

We found that cortical atrophy (i.e., cortical sulcus width ≥2.5 mm) was associated with a reduction in the brain volume and an increase in the CSF volume, CSF volume ÷ brain volume, and CSF volume ÷ intracranial volume. Multiple investigators have provided evidence that the cortical sulcus width increases with age, indicating the presence of brain atrophy [19, 23–25]. Additionally, other researchers have cited evidence that a cortical sulcus width similar to the value used in our study, ≥2.5 mm, indicates the presence of cortical atrophy [6, 12, 19, 26]. The aforementioned indicates that a cortical sulcus width ≥2.5 mm is a manifestation of cortical atrophy.

We established that composite atrophy (ventricular or cortical atrophy) was associated with a reduction in the brain volume and an increase in the CSF volume, CSF volume ÷ brain volume, and CSF volume ÷ intracranial volume. Other investigators have also utilized a combination of ventricular and sulcus dimensions as a sign of age-related brain atrophy [5, 27–29].

The multiple associations of the 1-D measurements with the 3-D values imply that both quantitative methods capture similar changes of brain and CSF structures with atrophy. However, the weak correlation coefficients indicate that the two measurements provide distinctive results. That is, 1-D measurements are not a complete quantitative replacement for 3-D interrogation. In particular, lateral ventricular body width principally reflects changes in that specific structure; whereas CSF volumes represent ventricle and extra-ventricle alterations. While 1-D measurements may be more practical, it is likely that 3-D computations might provide more relevant information, and thus insight, regarding certain aspects of brain atrophy.

### ICH associations

We showed that ICH was associated with 1-D (i.e., the ventricular width, ventricular width ÷ brain width, ventricular width ÷ intracranial width, central atrophy, cortical atrophy, and composite atrophy) and 3-D (i.e., the CSF volume, CSF volume ÷ brain volume, and CSF volume ÷ intracranial volume) manifestations of brain atrophy. Although brain atrophy has been promulgated as a risk for ICH [30–34], there was no evidence to support this notion, until our previous publication demonstrated an association with 1-D estimates of brain atrophy [1]. The current study provides additional evidence that ICH is associated with age-related brain atrophy, represented by intracranial 3-D measurements. To our knowledge, this is the first time that this relationship has been demonstrated.

It is important to note that ICH had no association with age, although the brain atrophy dimensions correlated with age. Of relevance, the correlation coefficients for age and atrophy manifestations in our study were only moderate to weak. This is exemplified in the study by Gur et al. which showed that advancing age had a negative correlation with the brain volume and a positive association with the CSF volume [13]. However, examination of their scatter plots indicated that there is substantial variance of brain and CSF volumes with each age range. It is likely that this variance is, in part, responsible for the correlation of ICH with brain atrophy, but not with age. That is, the existence of brain atrophy needs to be determined according to the individual’s brain imaging and is not an assumption based on age.

ICH progressively increased with central atrophy, cortical atrophy, and both entities and it had an independent association with central atrophy and cortical atrophy. These findings support the notion that the presence of brain atrophy should be predicated on the basis of both ventricular and cortical sulcus examination. The increased rates of ICH with major admission neurologic dysfunction and hospital mortality highlight the devastating effects that ICH can have on clinical outcomes.

### Ancillary associations with 1-D and 3-D measurements

In the current study, males were found to have increased brain width, intracranial width, brain volume, and intracranial volume, and decreased CSF volume ÷ intracranial volume, when compared to females. Other investigators have also found that males have increased intracranial width [6], brain volume [13, 16, 18], and intracranial volume [18], relative to females. These observations imply that decreases in female brain volume are likely related to cranial size.

The current investigation demonstrated that dementia patients had more cortical atrophy, reduced brain width, increased ventricular ÷ brain width, increased ventricular ÷ intracranial width, increased CSF volume, reduced brain volume, increased CSF volume ÷ brain volume, and increased CSF volume ÷ intracranial volume. Of relevance, the literature provides evidence that others have found that dementia is associated with increased cortical sulcal width [8, 35, 36], increases in lateral ventricular size [4, 35], increased lateral ventricular body width-to-intracranial width ratios [4, 8], increased ventricle-to-brain area ratio [36], and higher ventricle volume-to-cranial volume quotients [23]. The higher rate of dementia in females, in the current study, may help to explain the lower intracranial volume associated with dementia.

Patients with pre-admission anemia had a proclivity toward brain atrophy, when compared to those without anemia. We are uncertain as to the clinical rationale for this observation. Similarly, patients with history of cardiac disease or those receiving pre-admission anti-thrombotic agents had an association with increased brain atrophy. The grounds for these findings are also unclear.

The 3-month neurologic dysfunction (bad) outcome was associated with a manifestation of brain atrophy (increased CSF volume ÷ brain volume), pre-admission dementia, and major admission neurologic dysfunction. These observations seem to be clinically intuitive.

### 1-D study measurements, a practical method

Relevant to the primary 1-D brain atrophy measurements used in the current study, a representative summary of the literature is presented in Table 11. The methodological practicality for the current study measurements are considered in context of simplicity or complexity of other methods. Many of the other methods require multiple measurements; mandate that multiple cuts of the brain CT must be analyzed in order to determine the appropriate dimension to be used; or require special software. Relative to outcome associations, an indication of clinical validation, the 1-D measurements used in the current study had multiple significant relationships. Other methods typically had less support. Some of the other methodologies were only validated by a subjective interpretation that atrophy was present [2, 27], whereas others provided no evidence of validation in the manuscript [4, 7, 11]. We believe that the principal 1-D measurements used in the current study are practical, objective, and supported by their multiple associations with clinical outcomes and conditions.

**Table 11:** Complexity of 1-dimensional atrophy measurements and outcome associations

	No.	Methodological complexity:	Outcome associations:
LVB Width:			
Current investigation	177	S-C	age, ICH, CSF volume
Gonzalez, 1978 [2]	100	M-C (plus frontal horn width)	atrophy (subjective)
LVB ÷ IC Width:			
Current investigation	177	S-C	age, CSF ÷ brain volume, CSF ÷ IC volume, ICH, dementia, anemia
Earnest, 1979 [3]	59	S-C	age
Gado, 1983 [4]	24	S-C	none
Steiner, 1985 [5]	148	S-C	age
Sulcal Width:			
Current investigation	177	S-C	age, CSF volume, brain volume, CSF ÷ brain volume, CSF ÷ IC volume, ICH, dementia, cardiac history
Ford, 1981 [7]	59	M-C (largest sulcus)	none
Pirttila, 1992 [9]	416	M-C (subjective visual widening)	age, # medications, DM, HTN, CVA
Pasquier, 1996 [10]	50	M-C (13 areas; subjective widening)	none
Earnest, 1979 [3]	59	M-C (sum of 4-largest sulci)	age
Gyldensted, 1977 [6]	100	M-C (largest width)	age
Coyle, 2006 [11]	35	S-S (mean width)	none
Kochunov, 2008 [12]	31	S-S (3-D mean span)	age
Gonzalez, 1978 [2]	100	M-C (sum 4 largest on highest 3 cuts)	atrophy (subjective)
Kohlmeyer, 1983 [8]	150	M-C (sum of widest for frontal, temporal, parietal lobes)	age
Composite Atrophy:			
Current investigation	177	S-C (see LVB Width & Sulcal Width, above)	brain volume, CSF volume, CSF ÷ brain volume, CSF ÷ IC volume, ICH, cardiac history, anti-thrombotic agent use
Fox, 1975 [27]	35	M-C (sum LVB + frontal horn width; sum 4 largest sulci, highest 2 cuts)	atrophy (subjective)
Gonzalez, 1978 [2]	100	M-C (see above)	atrophy (subjective)

*LVB* lateral ventricular body, *S-C* single cut, *ICH* intracranial hemorrhage, *CSF* cerebrospinal fluid, *M-C* multiple cuts, *IC* intracranial, *DM* diabetes mellitus, *HTN* hypertension, *CVA* prior cerebrovascular accident, *S-S* special software

### Study limitations

Although the study was retrospective, the patients included in the study were identified through a reliable process, as described in our previous manuscript [1]. Patients’ ICH status was determined by the first-author after reviewing CT scans and reports for the study patients and the credentials of the first author were described in our previous manuscript [1]. Although the current study excluded some patients used in the original study that may have created potential bias, the similarity of the ICH rates mitigates this likelihood. While some may find fault with our 3-D computations using specific CSF and brain parenchymal Hounsfield ranges, similar correlations for the CSF volume ÷ brain volume and CSF volume ÷ intracranial volume (CSF volume + brain volume) with other outcomes suggest that the Hounsfield ranges were valid and without CSF volume and brain volume overlap. Hemorrhage may have confounded accurate brain volume computations, in those with ICH. However, the major of patients did not have ICH and, of those with ICH, the volumes were not extensive.

## Conclusions

The 3-D measurement method seems reasonable based on 1) multiple associations of the values with clinical conditions and outcomes; 2) similarity of results with those in the literature; and 3) examination of the highlighted CT images. The correlation of CT 1-D measurements with 3-D values indicates that the lateral ventricular body width and cortical sulcus width are reliable indicators of age-related brain atrophy. ICH is associated with CT 3-D indicators of brain atrophy, as well as 1-D manifestations. Multiple clinical conditions were also associated with the 1-D measurements. Together, this indicates that our 1-D criteria are valid, relative to clinical outcomes and conditions, and 3-D measurements of age-related brain atrophy. Results indicate that the presence of brain atrophy should be based on objective CT 1-D or 3-D measurements, and not only on the patient’s age. Because 3-D software is not available on many CT scanners or easy to use, brain atrophy assessment using 1-D measurement is typically more practical and available. Our radiology department is in the process of incorporating lateral ventricular body width >30 mm and cortical sulcus width ≥2.5 mm assessment into the radiologist’s routine brain CT examination for patients aged ≥60 years. The radiology report will include statements regarding the presence of age-related atrophy changes, when present, and central and cortical atrophy, when criteria are met. We believe that such patient risk stratification will assist in advancing hospital system process improvement and will assist healthcare providers in considering relevant clinical management and prevention strategies.

## Ethics approval

This study was approved by the Mercy Health Youngstown Institutional Review Board for human investigations (#14-003). The need for written informed consent from the patients was waived, because of the study’s retrospective nature. Patient records/information was anonymized and de-identified prior to analysis.

## Availability of supporting data

Due to statutory provisions regarding data- and privacy protection, the dataset supporting the conclusions of this article are only available upon individual request directed to the corresponding author.

## Abbreviations

CSF: Cerebrospinal fluid
CT: Computed tomography
DICOM: Digital imaging and communications in medicine
ICH: Intracranial hemorrhage
PACS: Picture archiving and communication system

## References

[1] Dunham CM, Hoffman DA, Huang GS, Omert LA, Gemmel DJ, Merrell R (2014). Traumatic intracranial hemorrhage correlates with preinjury brain atrophy, but not with antithrombotic agent use: a retrospective study. PLoS One.

[2] Gonzalez CF, Lantieri RL, Nathan RJ (1978). The CT scan appearance of the brain in the normal elderly population: a correlative study. Neuroradiology.

[3] Earnest MP, Heaton RK, Wilkinson WE, Manke WF (1979). Cortical atrophy, ventricular enlargement and intellectual impairment in the aged. Neurology.

[4] Gado M, Hughes CP, Danziger W, Chi D (1983). Aging, dementia, and brain atrophy: a longitudinal computed tomographic study. AJNR Am J Neuroradiol.

[5] Steiner I, Gomori JM, Melamed E (1985). Progressive brain atrophy during normal aging in man: a quantitative computerized tomography study. Isr J Med Sci.

[6] Gyldensted C (1977). Measurements of the normal ventricular system and hemispheric sulci of 100 adults with computed tomography. Neuroradiology.

[7] Ford CV, Winter J (1981). Computerized axial tomograms and dementia in elderly patients. J Gerontol.

[8] Kohlmeyer K, Shamena AR (1983). CT assessment of CSF spaces in the brain in demented and nondemented patients over 60 years of age. AJNR Am J Neuroradiol.

[9] Pirttila T, Jarvenpaa R, Laippala P, Frey H (1992). Brain atrophy on computerized axial tomography scans: interaction of age, diabetes and general morbidity. Gerontology.

[10] Pasquier F, Leys D, Weerts JG, Mounier-Vehier F, Barkhof F, Scheltens P (1996). Inter- and intraobserver reproducibility of cerebral atrophy assessment on MRI scans with hemispheric infarcts. Eur Neurol.

[11] Coyle TR, Kochunov P, Patel RD, Nery FG, Lancaster JL, Mangin JF, Riviere D, Pillow DR, Davis GJ, Nicoletti MA (2006). Cortical sulci and bipolar disorder. Neuroreport.

[12] Kochunov P, Thompson PM, Coyle TR, Lancaster JL, Kochunov V, Royall D, Mangin JF, Riviere D, Fox PT (2008). Relationship among neuroimaging indices of cerebral health during normal aging. Hum Brain Mapp.

[13] Gur RC, Mozley PD, Resnick SM, Gottlieb GL, Kohn M, Zimmerman R, Herman G, Atlas S, Grossman R, Berretta D (1991). Gender differences in age effect on brain atrophy measured by magnetic resonance imaging. Proc Natl Acad Sci U S A.

[14] Pfefferbaum A, Mathalon DH, Sullivan EV, Rawles JM, Zipursky RB, Lim KO (1994). A quantitative magnetic resonance imaging study of changes in brain morphology from infancy to late adulthood. Arch Neurol.

[15] Resnick SM, Pham DL, Kraut MA, Zonderman AB, Davatzikos C (2003). Longitudinal magnetic resonance imaging studies of older adults: a shrinking brain. J Neurosci.

[16] Akdogan I, Kiroglu Y, Onur S, Karabuluti N (2010). The volume fraction of brain ventricles to total brain volume: a computed tomography stereological study. Folia Morphol.

[17] Fjell AM, McEvoy L, Holland D, Dale AM, Walhovd KB (2013). Brain changes in older adults at very low risk for Alzheimer’s disease. J Neurosci.

[18] Matsumae M, Kikinis R, Morocz IA, Lorenzo AV, Sandor T, Albert MS, Black PM, Jolesz FA (1996). Age-related changes in intracranial compartment volumes in normal adults assessed by magnetic resonance imaging. J Neurosurg.

[19] Meese W, Kluge W, Grumme T, Hopfenmuller W (1980). CT evaluation of the CSF spaces of healthy persons. Neuroradiology.

[20] Blatter DD, Bigler ED, Gale SD, Johnson SC, Anderson CV, Burnett BM, Parker N, Kurth S, Horn SD (1995). Quantitative volumetric analysis of brain MR: normative database spanning 5 decades of life. AJNR Am J Neuroradiol.

[21] Takeda S, Matsuzawa T (1984). Measurement of brain atrophy of aging using X-ray computed tomography: sex difference in 1045 normal cases. Tohoku J Exp Med.

[22] Barron SA, Jacobs L, Kinkel WR (1976). Changes in size of normal lateral ventricles during aging determined by computerized tomography. Neurology.

[23] Turkheimer E, Cullum CM, Hubler DW, Paver SW, Yeo RA, Bigler ED (1984). Quantifying cortical atrophy. J Neurol Neurosurg Psychiatry.

[24] Kochunov P, Mangin JF, Coyle T, Lancaster J, Thompson P, Riviere D, Cointepas Y, Regis J, Schlosser A, Royall DR (2005). Age-related morphology trends of cortical sulci. Hum Brain Mapp.

[25] Drayer BP (1988). Imaging of the aging brain. Part I. Normal findings. Radiology.

[26] Ishii T (1983). A comparison of cerebral atrophy in CT scan findings among alcoholic groups. Acta Psychiatr Scand Suppl.

[27] Fox JH, Topel JL, Huckman MS (1975). Use of computerized tomography in senile dementia. J Neurol Neurosurg Psychiatry.

[28] Gado M, Patel J, Hughes CP, Danziger W, Berg L (1983). Brain atrophy in dementia judged by CT scan ranking. AJNR Am J Neuroradiol.

[29] Wu S, Schenkenberg T, Wing SD, Osborn AG (1981). Cognitive correlates of diffuse cerebral atrophy determined by computed tomography. Neurology.

[30] Holmes JF, Hendey GW, Oman JA, Norton VC, Lazarenko G, Ross SE, Hoffman JR, Mower WR (2006). Epidemiology of blunt head injury victims undergoing ED cranial computed tomographic scanning. Am J Emerg Med.

[31] Ellis GL (1990). Subdural hematoma in the elderly. Emerg Med Clin North Am.

[32] Hanif S, Abodunde O, Ali Z, Pidgeon C (2009). Age related outcome in acute subdural haematoma following traumatic head injury. Ir Med J.

[33] Gangavati AS, Kiely DK, Kulchycki LK, Wolfe RE, Mottley JL, Kelly SP, Nathanson LA, Abrams AP, Lipsitz LA (2009). Prevalence and characteristics of traumatic intracranial hemorrhage in elderly fallers presenting to the emergency department without focal findings. J Am Geriatr Soc.

[34] Doherty DL (1988). Posttraumatic cerebral atrophy as a risk factor for delayed acute subdural hemorrhage. Arch Phys Med Rehabil.

[35] Bird JM (1982). Computerized tomography, atrophy and dementia: a review. Prog Neurobiol.

[36] Wurthmann C, Bogerts B, Falkai P (1995). Brain morphology assessed by computed tomography in patients with geriatric depression, patients with degenerative dementia, and normal control subjects. Psychiatry Res.

